# Comparative Study between Ethanolic and *β*-Cyclodextrin Assisted Extraction of Polyphenols from Peach Pomace

**DOI:** 10.1155/2018/9491681

**Published:** 2018-03-08

**Authors:** Nada El Darra, Hiba N. Rajha, Espérance Debs, Fatima Saleh, Iman El-Ghazzawi, Nicolas Louka, Richard G. Maroun

**Affiliations:** ^1^Faculty of Health Sciences, Beirut Arab University, Tarik El Jedidah, Riad El Solh, P.O. Box 115020, Beirut 1107 2809, Lebanon; ^2^Unité de Recherche Technologies et Valorisation Agro-Alimentaire, Centre d'Analyses et de Recherche, Faculté des Sciences, Université Saint-Joseph, Riad El Solh, BP 11-514, Beirut 1107 2050, Lebanon; ^3^Faculty of Sciences, Department of Biology, University of Balamand, Koura, Lebanon

## Abstract

Peach byproducts are often regarded as food waste despite their high content in health-promoting components. Amongst the latter, polyphenols are bioactive molecules with significant health benefits. The present study investigated an eco-friendly and cost-effective method using a GRAS food additive, *β*-cyclodextrin (*β*-CD), for the recovery of polyphenols from peach pomace. *β*-CD assisted extraction of polyphenols was compared to that of conventional solvent (ethanol) extraction at the same concentrations (10 mg/mL, 20 mg/mL, 30 mg/mL, 40 mg/mL, and 50 mg/mL) in terms of quality (antiradical activity) and quantity. The extract obtained by 50 mg/mL *β*-CD assisted extraction showed the highest polyphenol (0.72 mg GAE/g DM) and flavonoid (0.35 mg catechin/g of DM) concentrations as maximal antiradical activity (6.82%) and a noted antibacterial activity. Our results showed the competitiveness of *β*-CD assisted extraction to recover a high quantity and quality of polyphenols from peach pomace suggesting *β*-CD as a green alternative method for phenolic extraction.

## 1. Introduction

Byproducts and waste obtained from food processing represent a major disposal problem for the food industry. A large number of these products are generated at different stages of the food supply chain of either vegetable or animal commodities [1]. Currently, waste management bodies are highly recommending that industrialists invest in new end-uses for such food byproducts. Valorisation of food waste sources is therefore becoming a prime interest, owing to their environmental and economic values. For instance, natural oils extracted from fruit seeds and kernels are used in cosmetics and pharmaceutical industry [2].

Peach (*Prunus persica* L.) is a fruit rich in polyphenols that are mainly localized in the pulp and peel tissues. Chlorogenic acid, catechin, epicatechin, rutin, and cyanidin-3-glucoside represent the main phenolic compounds of this fruit [3]. Although it is rich in ascorbic acid and carotenoids, it was found that the phenolic content of the peach is the major contributor to the observed antioxidative activity [4]. It is noteworthy to mention that, in China (mainland), 10^5^ MT (Metric Ton) of peach pomace has been at least estimated to be produced annually from peach juice processing [5].

In the present study, we are interested in the recovery of polyphenols from the peach pomace, as the studies related to peach polyphenols are very limited in the literature. Adil and coworkers [6] have optimized the subcritical extraction of phenolic content from peach pomace which was performed by selecting the pressure between 20 and 60 MPa, temperature between 40 and 60°C, ethanol concentration at 14–20 wt.%, and extraction time from 10 to 40 min on subcritical (CO_2_ + ethanol) extraction of polyphenol. The total phenolic content from peach pomace was 0.26 mg gallic acid equiv./g and the antiradical efficiency was 1.5 mg 1,1-diphenyl-2-picrylhydrazyl (DPPH)/mg [6].

Traditionally, extraction of phenolic compounds from natural resources is carried out using organic solvents, for example, methanol, ethanol, and acetone [7]. However, these extraction processes are quite laborious and involve large amounts of solvents associated with serious environmental issues, thus limiting their application. The need to develop new and cost-effective methods used to extract high levels of polyphenols with enhanced bioavailability from products such as peach turns out to be urgent. There is an increasing demand in recent years for cheaper, safer, and eco-friendly alternatives to organic solvents. Cyclodextrins (CDs) based extraction is an emerging “green” technology of great potential. CDs are naturally occurring cyclic oligosaccharides arising from the degradation of starch and are FDA “Food and Drug Administration” approved [8]. They appear as *α*-, *β*-, and *γ*-CDs, knowing that *β*-CDs are the least expensive and the most widely used [9]. *β*-CDs are increasingly employed as encapsulating agents for plants bioactive molecules such as polyphenols, hence preserving their biological properties, extending their shelf life, and protecting them away from environmental factors (light, temperature, oxidation, pH, and moisture) [9–11]. Compared to organic solvents, *β*-CDs assisted extraction is more economic, safe, and green [12].

A recent study conducted by Rajha and collaborators [13] has clearly shown the capacity of *β*-CD to extract polyphenols from vine shoots with a higher radical scavenging capacity compared to conventional ethanol extraction. Similarly, Ratnasooriya and Rupasinghe [14] demonstrated that the assisted recovery of total phenolic compounds from grape pomace slurry using *β*-CD at room temperature was significantly higher compared to water extraction [14]. Diamanti and coworkers [15] also reported that green extraction using *β*-CD enhanced the total phenolic content and the radical scavenging activity of whole pomegranate extracts [15].

To the best of our knowledge, there are no available studies on the use of CDs in recovering polyphenols from peach pomace. The aim of this study was to compare the efficiencies of polyphenols extraction using *β*-CD, aqueous and organic solvents. The extraction processes were optimized by monitoring solvent concentration, temperature, solvent volume to sample ratio, and extraction time. The resulting extracts were then analysed for total phenolic content, flavonoids, tannins, vitamin C, and carotenoids contents. In addition, these compounds were examined for their antioxidant and antimicrobial activity.

Finally, high-performance liquid chromatography (HPLC) was implemented to identify the phenolic components present in every extract.

## 2. Materials and Methods

### 2.1. Samples Preparation and Dry Matter Content

Peach (*Prunus persica* L.) pomace was obtained from Conserves Modernes Chtaura (Chtaura, Lebanon) specialized in the production of jams and purees. The pomace consists of pressed skins and pulp residue. The dry matter content for the raw material was determined by weighing an appropriate amount of sample and drying it for 24 hours in a ventilated oven at 105°C [16].

### 2.2. Chemicals

All chemicals were purchased from Fluka Chemie GmbH (Buchs, Switzerland) or from Sigma-Aldrich (Steinheim, Germany).

### 2.3. Bacterial Strains, Culture Media, and Growth Conditions

Sixteen stock isolates of bacterial strains were used. They are clinical isolates obtained from previous research studies carried out in the Faculty of Medicine of Alexandria University, Egypt. The isolates included two strains of each of the following: Methicillin-resistant* Staphylococcus aureus* (MRSA), Methicillin-resistant* Staphylococcus epidermidis *(MRSE), coagulase-negative staphylococci,* Staphylococcus aureus*, high-level aminoglycoside-resistant enterococci (HLAR) (*Enterococcus faecalis* and* Enterococcus faecium *which were also resistant to vancomycin),* Pseudomonas aeruginosa*, and* Escherichia coli*. One strain of* Klebsiella pneumoniae* and* Acinetobacter baumannii* was also studied. The two strains of* Escherichia coli*,* Klebsiella pneumoniae*, and* Acinetobacter baumannii* were previously proved to be extended-spectrum *β*-lactamases (ESBL), by double-disc synergy and combined-disc tests.

### 2.4. Solid-Liquid Extraction Process

The extraction process of polyphenols from peach pomace was performed with a solid-liquid ratio of 1 : 10 (w/v). After *β*-CD aqueous extraction at different concentrations (10, 20, 30, 40, and 50 mg/mL) and ethanol extraction at different concentrations (10, 20, 30, 40, 50, and 500 mg/mL), the extracts were centrifuged at 5000 rpm (rotation per minute) for 15 min. *β*-CD aqueous concentrations were prepared by dissolving the required weight of *β*-CD in the specific water volume in a 50°C water bath while stirring for 2 hours of diffusion time [13].

### 2.5. Total Phenolic Content Determination: Folin-Ciocalteu Method

The total phenolic content was determined according to Folin-Ciocalteu (FC) method [17]. 0.2 mL of standard (gallic acid) or diluted sample, 1.0 mL of FC reagent, and 0.8 mL of Na_2_CO_3_ solution (7.5%) were mixed and allowed to stand for 2 hours at room temperature. Light absorption was measured at 750 nm by a spectrophotometer UV-VIS against a blank similarly prepared, but containing distilled water instead of extract. The total phenolic content (*Y*_*e*_) was expressed in grams of gallic acid equivalent (GAE) per gram of dry matter (DM) (g GAE/g DM).

### 2.6. Determination of Tannin Concentration

Total tannin content (g/L) and HCl index which represents the tannin polymerization degree were determined according to Ribérau-Gayon and collaborators [18]. Total tannin assay is based on the heating process of tannins in acidic medium leading to the formation of cyanidins. Two tubes were prepared, each containing 1 mL of diluted peach pomace extract, 0.5 mL of water, and 1.5 mL of 12 N HCl. The first tube was mixed and heated in a water bath at 100°C for 30 min. The second was kept at room temperature. Following the rapid cooling, 0.25 mL of ethanol was added to the mixture and the resulting absorbance was recorded at 520 nm.

The tannin concentration was calculated as follows:

(1) Tannin  concentrationmg/L=19.33×Δ  optical  densities.

### 2.7. Free Radical Scavenging Activity

The 1,1-diphenyl-2-picrylhydrazyl (DPPH) radical was used in the present study for the screening of the radical scavenging activity of the extracts [19]. The DPPH radical scavenging activity was measured using the spectrophotometer UV-VIS (Libra S32, Biochrom, France). The samples were tested at a concentration of 20 mg/mL and then mixed with 1000 *μ*L of 0.1 mM DPPH-ethanol solution and 450 *μ*L of 50 mM Tris-HCl buffer (pH 7.4). Methanol (50 *μ*L) was used for blank measurements in this experiment. After 30 min of incubation at room temperature, the reduction of the DPPH free radical was measured by reading the absorbance at 517 nm. Butylhydroxytoluene (BHT) (a synthetic antioxidant) was used as a positive control. The inhibition ratio (percent) was calculated according to the following equation:

(2) %  inhibition=absorbance  of  control−absorbance  of  test  sampleabsorbance  of  control∗100.

### 2.8. Determination of Total Flavonoids (TF)

The total flavonoids (TF) assay was conducted as previously described by Zhuang and coworkers [20] with some modification [20]. A volume of 1 mL of diluted extract or standard solution of catechin was placed in a 10 mL volumetric flask already containing 4 mL of H_2_O. Five minutes later, 0.3 mL of NaNO_2_ (5%) and 1.5 mL of AlCl_3_ (2%) were added. The mixture was shaken for 5 min, then 2 mL of 1 M solution of NaOH was added, and the mixture was well shaken again. The absorbance was measured at 510 nm against the blank. The results were calculated according to the calibration curve for catechin (*R*^2^ = 0.99). The content of TF was expressed as mg of catechin equivalent (CE) per g of dry matter content.

### 2.9. Vitamin C Analysis

Vitamin C estimation was done according to the Folin-Ciocalteu method [21]. Peach pomace extracts (0.2 mL) were added to 0.8 mL of 10% trichloroacetic acid and then well shaken. The mixtures were kept on ice for 5 min and then centrifuged at 3000*g* for 5 min. The extract was then diluted (1/10). Folin-Ciocalteu was diluted (1/10) and then 0.2 mL was added to the mixture and vigorously shaken. After 10 min, at room temperature, the absorbance was measured at 760 nm against distilled water as a blank and vitamin C was estimated through the calibration curve of the ascorbic acid standard.

### 2.10. *β*-Carotene Estimation

A simple UV spectrophotometric method was used for the analysis of *β*-carotene. The extraction of *β*-carotene was simply assessed on the liquid extracts by UV absorbance which was measured at 461 nm. The following equation was applied to determine the *β*-carotene concentration: *y* = 0.1069*x* − 0.0057, where *x* and *y* are, respectively, the *β*-carotene concentration in mg/L and UV absorption at 461 nm [22].

### 2.11. Determination of Minimum Inhibitory Concentration (MIC)

The broth microdilution method was used in a sterile 96-well microtiter plate (U shaped base) [23].

#### 2.11.1. Preparation of the Bacterial Inocula for MIC

Glycerol broth stocks were subcultured on a freshly prepared blood agar plate, incubated at 37°C overnight. Using a sterilized loop, five colonies of each strain were inoculated in 3 mL of cation adjusted Mueller Hinton broth, and the turbidity was compared to 0.5 McFarland standard. 1/100 dilution of this 0.5 McFarland was prepared to be used for the MIC.

#### 2.11.2. Peach Phenolic Extracts Preparation for MIC Assessment

The peach ethanolic extracts at different concentrations (10, 20, 30, 40, 50, and 500 mg/mL) were subjected to drying by rotary evaporator, in order to remove the ethanol. Afterwards, five serial dilutions, in sterile distilled water, of each of the following extracts were prepared: aqueous extract, *β*-cyclodextrin extract (50 mg/mL), ethanolic extract (50 mg/mL), and ethanolic extract (500 mg/mL) until reaching the concentrations 53 *μ*g/mL, 26 *μ*g/mL, 13 *μ*g/mL, 6 *μ*g/mL, and 3 *μ*g/mL. After the addition of equal volumes (100 *μ*L) of each concentration of these extracts to the bacterial strains (100 *μ*L) to be tested in each well of the plate, the final concentrations of the different phenolic extracts were reduced to 26 *μ*g/mL, 13 *μ*g/mL, 6.5 *μ*g/mL, 3.25 *μ*g/mL, and 1.6 *μ*g/mL. Four microtiter plates were used in this experiment. After overnight incubation at 37°C, all the plates were examined for the MIC of each of the phenolic extracts concentration that inhibits the bacterial growth. All the peach phenolic extracts were filter sterilized using 0.4 *μ*m disposable syringe filters prior to the assessment.

### 2.12. High-Performance Liquid Chromatography with Diode Array Detection (HPLC-DAD) Analysis

Polyphenol analyses were performed using a Jasco HPLC system (Japan) (PV-2089) equipped with an autosampler, an L-2130 pump, a Jetstream column oven, and an L-2450 diode array detector. The separation was carried out with a Column C18 25 × 0.46 mm (Teknokroma Professional Friendly Lichrospher 100 RP18 5 *μ*M, 25 × 0.46, serial number NF-21378, Barcelona, Spain), using a gradient elution at a flow rate of 1 mL per min for 30 min. Trans-cinnamic acid, caffeic acid, hydroxybenzoic acid, chlorogenic acid, catechin, rutin, quercetin, protocatechin, gallic acid, epigallocatechin, kaempferol, catechin gallate, and myricetin standards were used for identification and quantification purposes with HPLC-DAD, respectively. The mobile phase consisted of acidified nanopure water at pH 2.3 with HCl (A) and acetonitrile HPLC grade (B). The elution was isocratic conditions from 0 to 5 min with 85% A and 15% B. Gradient from 5 to 30 min began with 85% A and 15% B and ended with 0% A and 100% B followed by isocratic conditions from 30 to 35 min with 0% A and 100% B to reequilibrate the column. The injection volume was 10 *μ*L. The identification of peaks was based on retention time and the spectra of external standards. The concentration of phenolic compounds was determined from standard curves constructed for individual compounds by injecting different concentrations of corresponding standards [24].

### 2.13. Statistical Analysis

Each experiment was conducted in duplicate and analysis repeated twice. Means and standard deviations of data were calculated. The error bars in all figures correspond to the standard errors. Variance analyses (ANOVA) and Least Significant Difference (LSD) test were conducted to evaluate the significant differences between the results. STATGRAPHICS® Centurion XV (StatPoint Technologies, Inc.) was used to perform statistical analysis.

## 3. Results and Discussion

### 3.1. Effect of Ethanolic and Beta-Cyclodextrin Assisted Extractions on the Phenolic Content, Tannins, and Flavonoids of the Peach Pomace Extract

**Figure 1:**
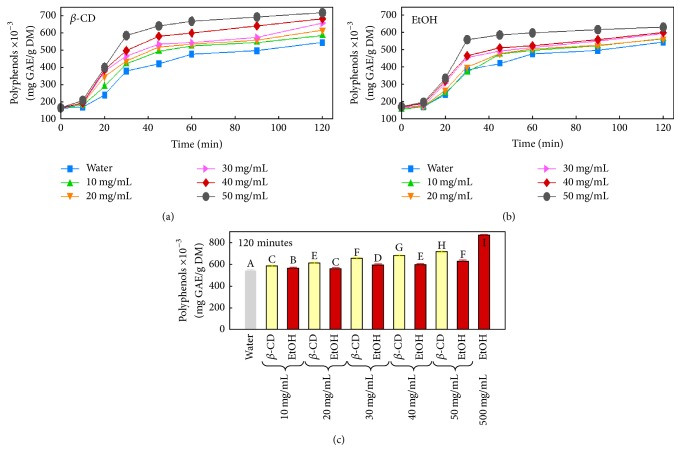
(a) Extraction kinetics of polyphenols with different concentrations (0, 10, 20, 30, 40, and 50 mg/mL) of beta-cyclodextrin (*β*-CD) and (b) different concentrations (0, 10, 20, 30, 40, and 50 mg/mL) of ethanol (EtOH). (c) Comparison of polyphenol recovery with both solvents (*β*-CD and EtOH) at the same concentrations after 120 minutes of extraction. Different superscript letters indicate significant statistical difference (*p* < 0.05).

Figure 1 shows the recovery of polyphenols from peach pomace by solid-liquid extraction using *β*-CD or EtOH solvents. Many parameters affect the solid-liquid extraction process. One of these parameters is the extraction time that had to be studied before undertaking the trials. For this purpose, a systematic study, between 0 and 120 minutes, was conducted for the recovery of total phenolic content (TPC) using the three extraction media: water, beta-cyclodextrin, and ethanol at different concentrations. In Figures 1(a) and 1(b), we noticed that the longer the extraction time, the better the TPC yield. Moreover, a closer observation of the plots revealed a common kinetics pattern of extraction for the three solvents. All the kinetics followed a classical three-step model. A slight increase in the TPC yield was observed during the first ten minutes of the extraction process. Less than 20% increase was recorded for the best datum. Then a second step occurred where a sharp rise of the extraction efficiency is clearly noticed. The TPC yield was around 3-fold higher at 30 min compared to 10 min. Finally, the slope decreased significantly between 30 and 120 min where the recovery capacity had plateaued out with barely 30% of improvement. To stretch out the argument, this model could strongly reflect what is happening at the microscopic level (Figure 1(c)). Step one marks the time needed by the solvent to diffuse inside the cellular structure and to prepare the biological material for the subsequent step. Step two represents the highest extraction efficiency of the cellular content in TPC. Step three witnesses an almost stabilization of the extraction yield, which means that the three solvents would have reached their maximum capacity of extraction under those conditions rather than unveiling the maximum cellular content in TPC.

On a different note, it is noteworthy to mention that the effect of the concentration of both solvents *β*-CD and ethanol was directly proportional to the TPC yield. The tested solvents were used over a concentration range of 10 to 50 mg/mL; the higher the concentration, the better the TPC yield. Water was significantly the least efficient in TPC extraction.

The increase in both *β*-CD and EtOH concentrations up to 50 mg/mL significantly (*p* < 0.05) enhances polyphenol extraction compared to water solvent (Figures 1(a) and 1(b)). However, at the same concentrations, *β*-CD is more efficient than the organic solvent EtOH. For example, at 50 mg/mL of both solvents, polyphenol yields were 715 and 630 mg GAE/g DM for *β*-CD and EtOH, respectively. The highest polyphenol concentration was obtained with 500 mg/mL EtOH (865 mg GAE/g DM). However, compared to 50 mg/mL EtOH, the concentration was enhanced by only 1.2 times with an increase of tenfold EtOH concentration.

**Figure 2:**
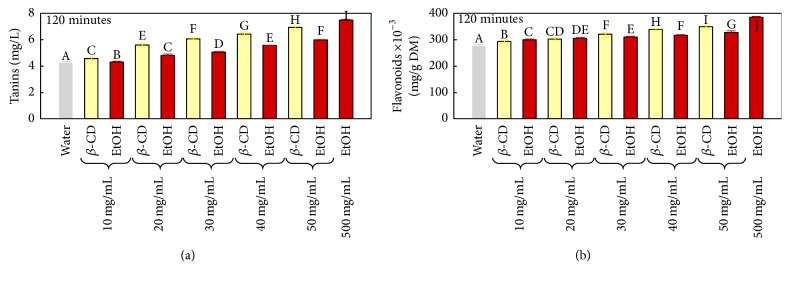
Comparison of (a) tannins and (b) flavonoids recovery using *β*-CD and EtOH at the same concentrations after 120 minutes of extraction. Different superscript letters indicate significant statistical difference (*p* < 0.05).

In concordance with total polyphenol extraction, tannins and flavonoids recovery from peach pomace was also enhanced by *β*-CD and EtOH compared to the aqueous extraction. At all the studied concentrations (10, 20, 30, 40, and 50 mg/mL), *β*-CD was more efficient than EtOH (Figures 2(a) and 2(b)). *β*-CD efficacy compared to water is likely due to the inclusion complexes it forms with bioactive molecules, which increases their solubility and therefore their recovery (Szente, L. et al., 2004, [9]). On the other hand, ethanol efficiency is rather related to the alteration it causes to the cellular membranes which increases their permeability and therefore the diffusion process of intracellular components [25].

### 3.2. Effect of Ethanolic and Beta-Cyclodextrin Assisted Extraction on Beta-Carotene and Vitamin C Content of the Peach Pomace Extract

**Figure 3:**
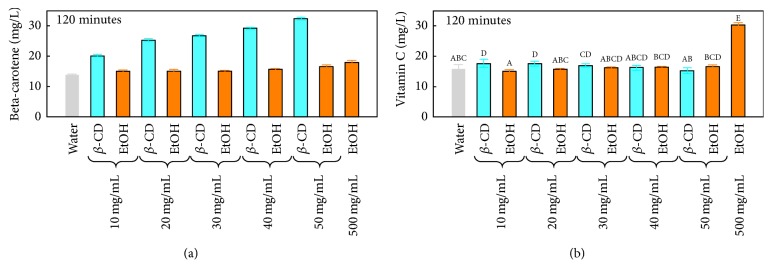
Comparison of (a) beta-carotene and (b) vitamin C recovery using *β*-CD and EtOH at the same concentrations after 120 minutes of extraction. Different superscript letters indicate significant statistical difference (*p* < 0.05).

Figure 3 shows the recovery of beta-carotene and vitamin C from peach pomace with increasing *β*-CD and EtOH concentrations. *β*-CD ameliorates by far the extraction of beta-carotene compared to EtOH. For example, 50 mg/mL of *β*-CD permits the obtainment of a better beta-carotene yield (32 mg/L) than 500 mg/mL of EtOH (18 mg/L). In contrast, vitamin C extraction was not enhanced (16 mg/L) by *β*-CD or EtOH addition up to 50 mg/mL. This result is in agreement with the study of Navarro et al., 2011, who showed that the addition of *β*-CD produces low or null effect on the vitamin C content of pasteurized orange juice. The highest vitamin C yield (30 mg/L) was obtained with 500 mg/mL of EtOH.

### 3.3. Antiradical Activity of the Peach Pomace Extracted by Ethanolic and Beta-Cyclodextrin Assisted Extraction

**Figure 4:**
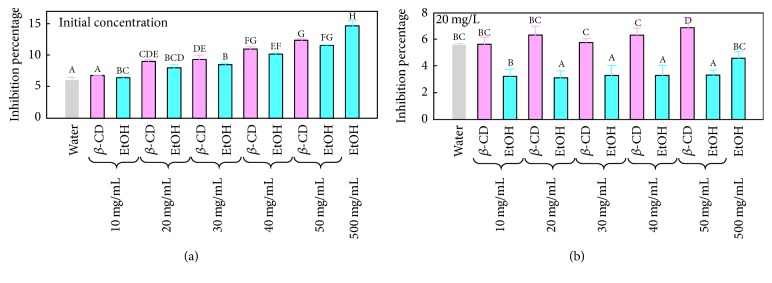
Comparison of the radical scavenging capacity of *β*-CD and EtOH extracts (a) at their initial concentration and (b) at 20 mg/mL of polyphenols. Different superscript letters indicate significant statistical difference (*p* < 0.05).

Since no amelioration of vitamin C extraction was observed in water, *β*-CD, and EtOH concentrations up to 50 mg/mL (Figure 3(b)), the radical scavenging capacity of those extracts was therefore attributed to their polyphenol and beta-carotene contents. This was in accordance with the study of Cantín et al., 2009, who showed no correlation between vitamin C and antiradical activity [26].  Figure 4(a) shows the inhibition percentage of the DPPH radical obtained with the different extracts at their initial polyphenol concentrations shown in Figure 1. The higher the polyphenol concentration in the extracts, the better their antiradical capacity was. Many authors showed the concentration-dependent radical scavenging activity of polyphenol extracts [27, 28]. At the same polyphenol concentration (Figure 4(b)), all *β*-CD extracts showed better antiradical capacity than those obtained with EtOH suggesting a better quality of the recovered molecules. The efficiency of *β*-CD in terms of polyphenol extraction and the enhancement of their radical scavenging capacity was also shown on vine shoots [13]. Similar study showed that microencapsulation of Mexican oregano essential oils with *β*-cyclodextrin enhanced their antiradical activity [29]. This is attributed to the protection effect of polyphenol encapsulation by *β*-CD since it is likely to preserve them from heat-degradation, UV light, and oxidation [30].

### 3.4. Antibacterial Activity of the Peach Pomace Extracted by Ethanolic and Beta-Cyclodextrin Assisted Extraction

The antimicrobial activities of the three peach pomace extracts (*β*-CD 50 mg/mL, EtOH 50 mg/mL, and EtOH 500 mg/mL) were tested against different Gram-positive and Gram-negative bacterial strains (Table 1) using MIC at different concentrations 1.6, 3.25, 6.5, 13, and 26 *μ*g/mL. The ethanol extract (500 mg/mL) of peach pomace showed the highest inhibitory activity against the different tested Gram-positive and Gram-negative strains. Our results are in accordance with the study by Zarai et al. [31] who showed that ethanolic extracts were more effective against both Gram-positive and Gram-negative bacteria tested, which could be explained by a better extraction of phenolic and flavonoid components at ethanolic concentration of 500 mg/mL [31]. This could be probably due to the fact that ethanol at a high concentration of 500 mg/mL is more selective in extracting polyphenols with higher biological activity. This was consistent with our radical scavenging activity and flavonoids findings, where ethanol 500 mg/mL showed the higher inhibitor activity of DPPH with high flavonoids content. Other extracts (*β*-CD 50 mg/mL, EtOH 50 mg/mL) were found to be active against all the tested species of Gram-positive bacteria whereas the Gram-negative bacteria remained unaffected. Our results are in agreement with the findings of Naz et al. [32] who showed that phenolic compounds have enhanced activity against Gram-positive strains compared to Gram-negative [32], due to the presence of an outer membrane in the cell wall of Gram-negative strains acting as permeability barrier and thus reducing the uptake [33]. Compared to EtOH 50 mg/mL, *β*-CD 50 mg/mL in water showed lower MIC for the different Gram-positive strains, which leads to a higher antibacterial activity. The ethanol was used to be compared at the same concentration as *β*-CD, since it is a solvent with relatively low toxic potential, and use of ethanol was permitted in the food industry [34]. The recovery of polyphenols possessing a higher antibacterial activity was noted for *β*-CD 50 mg/mL assisted extraction comparing to EtOH 50 mg/mL. This could be due to a better encapsulation and activity of polyphenols in *β*-CD 50 mg/mL, leading to a higher antibacterial activity.

### 3.5. Polyphenol Quantification by High-Performance Liquid Chromatography

**Figure 5:**
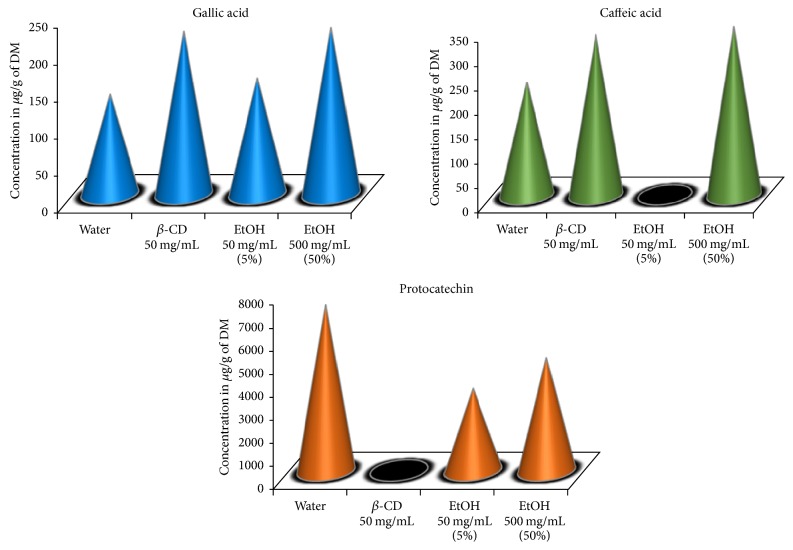
Gallic acid, caffeic acid, and protocatechin content of water, *β*-CD (50 mg/mL), EtOH (50 mg/mL), and EtOH (500 mg/mL) extracts.

Polyphenol quantity and diversity were also determined by HPLC on water, *β*-CD (50 mg/mL), and EtOH (50 and 500 mg/mL) extracts (Figure 5). *β*-CD selectively enhances the extraction of gallic and caffeic acids with yields equal to 220 and 328 *μ*g/g DM, respectively. 500 mg/mL of EtOH was required to reach the same gallic and caffeic acids' yields. The higher concentrations of these phenolic acids in *β*-CD (50 mg/mL) and EtOH (500 mg/mL) could explain their high antibacterial activities against the bacterial strains stated above [35], as well as their high scavenging activity against the DPPH radical [36]. However, water seems to better extract protocatechin (7188 *μ*g/g DM) compared to EtOH 50 mg/mL (3561 *μ*g/g DM) and EtOH 500 mg/mL (4899 *μ*g/g DM).

## 4. Conclusion

Our study demonstrated that *β*-CD assisted extraction of peach pomace enhanced the phenolic content, tannins, flavonoids, carotenoids, antiradical, and antimicrobial activities compared to organic solvent extraction. Our study verified that encapsulation of peach pomace polyphenols in *β*-CD is efficient in terms of the quantity and quality of the extracted molecules. The use of *β*-CD in an assisted extraction process is a green technology for food waste recovery.

## Figures and Tables

**Table 1:** Minimum inhibitory concentration (*µ*g/mL) of different Gram + and Gram − bacteria obtained with *β*-CD 50 mg/mL, EtOH 50 mg/mL, and EtOH 500 mg/mL extracts.

Bacteria/POMs	Minimum inhibitory concentration (*µ*g/mL)		
	*β*-CD 50 mg/mL	EtOH 50 mg/mL	EtOH 500 mg/mL
Methicillin-resistant *Staphylococcus aureus* (MRSA1) (Gram +)	13	26	13
Methicillin-resistant *Staphylococcus aureus* (MRSA2) (Gram +)	26	-	1.5
Methicillin-resistant *Staphylococcus epidermidis* MRSE 1297 (Gram +)	-	-	1.5
Methicillin-resistant *Staphylococcus epidermidis* MRSE 1296 (Gram +)	-	-	6.5
Coagulase-negative staphylococci 1664 (Gram +)	-	-	13
Coagulase-negative staphylococci 1530 (Gram +)	13	-	13
*Staphylococcus aureus* 1966 (Gram +)	6.5	13	13
*Staphylococcus aureus* 2030 (Gram +)	3	13	13
High-level aminoglycoside-resistance enterococci HLAR (Gram +)	13	-	13
Vancomycin-resistant enterococci VRE (Gram +)	13	-	1.5

*Pseudomonas aeruginosa *27 (Gram −)	-	-	1.5
*Pseudomonas aeruginosa *32 (Gram −)	-	-	1.5
*Escherichia coli *1238 (Gram −)	-	-	13
*Escherichia coli *1250 (Gram −)	-	-	13
*Klebsiella pneumoniae *184 (Gram −)	-	-	6.5
*Acinetobacter baumannii *1204 (Gram −)	-	-	1.5
